# Direct electrochemical generation of organic carbonates by dehydrogenative coupling

**DOI:** 10.3762/bjoc.14.135

**Published:** 2018-06-27

**Authors:** Tile Gieshoff, Vinh Trieu, Jan Heijl, Siegfried R Waldvogel

**Affiliations:** 1 Institute of Organic Chemistry, Johannes Gutenberg University Mainz, Duesbergweg 10-14, 55128 Mainz, Germany; 2 Graduate School Materials Science in Mainz, Staudingerweg 9, 55128 Mainz, Germany; 3 Covestro AG, Kaiser-Wilhelm-Allee 60, 51373 Leverkusen, Germany; 4 Covestro NV, Haven 507 - Scheldelaan 420, 2040 Antwerpen, Belgium

**Keywords:** anode, boron-doped diamond, dehydrogenative coupling, electrochemistry, organic carbonates

## Abstract

Organic carbonates are an important source for polycarbonate synthesis. However, their synthesis generally requires phosgene, sophisticated catalysts, harsh reaction conditions, or other highly reactive chemicals. We present the first direct electrochemical generation of mesityl methyl carbonate by C–H activation. Although this reaction pathway is still challenging concerning scope and efficiency, it outlines a new strategy for carbonate generation.

## Introduction

**Figure 1:**
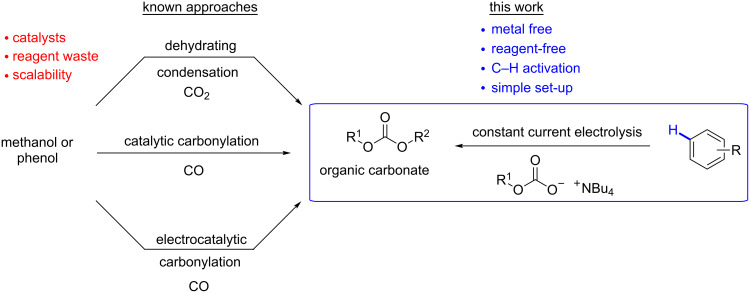
Current non-phosgene approaches to organic carbonates.

Polycarbonates are high-performance polymeric materials with versatile applications in various fields with economic impact, e.g., construction, food, and pharmaceutical industry [1]. For their technical large-scale production, organic carbonates like diphenyl carbonate (DPC) or dimethyl carbonate (DMC) are key intermediates. Processes for the carbonate generation have been investigated since the 1950s [2]. Although the use of these starting materials is straightforward and unobjectionable at first sight, their generation usually requires highly reactive chemicals. This comes with disadvantages in high safety requirements for handling these chemicals, such as ethylene oxide and phosgene [3]. Alternative approaches to carbonate generation are oxidative carbonylations or dehydrative condensations based on alcohols as starting materials (Figure 1) [4–6]. However, both alternatives do not compete with the phosgene approach, since catalyst, excessive amounts of reagents, or harsh reaction conditions are necessary and provide rather low yields. Contemporary research also focuses on the incorporation of carbon dioxide by catalytic polymer formation with less reactive epoxides (other than ethylene oxide) [7–10].

Electrochemistry has the capability to access products by extraordinary reaction pathways. Electric current is an inexpensive reagent and inherently safe reaction set-ups ensure a resource saving and applicable technology [11–12]. Several groups developed interesting protocols to use electrochemistry for carbonate generation, but these approaches suffer from complex electrolysis set-ups and lack in scalability [13–20].

In this context, we decided to focus onto a novel electrochemical method for the generation of organic carbonates using inexpensive starting materials without the necessity of catalysts. Generally, alcohols serve as starting materials for the DPC and DMC synthesis. However, efficiency increases if non-functionalized aromatic compounds serve as feedstock. Boron-doped diamond (BDD) as electrode material has the capability to convert simple aromatic systems by direct C–H activation [21–27]. In contrast, other typical anode materials such as graphite, glassy carbon, or platinum tend to lead to electrode fouling when applying high positive potentials [28–30]. In combination with easily accessible carbonate sources, we tried to establish a new dehydrogenative approach to organic carbonates. Here, the study on the first direct electrochemical generation of organic carbonates by dehydrogenative coupling is presented.

## Results and Discussion

**Scheme 1:**
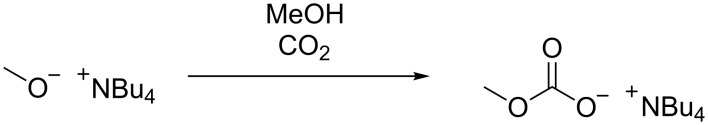
Preparation of tetrabutylammonium methyl carbonate by direct carbon dioxide incorporation.

Within initial experiments the anodic electrolysis of benzene in an aqueous media with metal carbonate salts was investigated. Due to the challenging combination of both, benzene and carbonate source in sufficient concentration within the electrolyte, first experiments were not successful. Benzene exhibits a solubility in water of 1.74 g/L [31]. However, upon addition of carbonates this solubility decreased to trace levels. Moreover, twofold functionalization of carbonate salts is challenging, because the stability of mono-functionalized intermediates is questionable. Therefore, we switched to an organic acetonitrile-based electrolyte system. Acetonitrile tolerates highly positive potential regimes, which are necessary for C–H activation of non-functionalized arenes. Since simple metal-based carbonate salts are not sufficiently soluble in organic media, the choice of carbonate source is crucial. Therefore, tetrabutylammonium methyl carbonate was employed. The solubility in acetonitrile is attributed to the tetrabutylammonium counterion. Its preparation is very simple by direct treatment of carbon dioxide with the methoxide alkylammonium salt (Scheme 1) [32]. Since this carbonate source is blocked at one end, the mono-functionalization is sufficient for product generation. Although mixed carbonates will be generated with this carbonate source, the products are also applicable in polycarbonate synthesis, as current non-phosgene diphenyl carbonate technology employs a disproportionation of such mixed carbonates [33].

**Scheme 2:**
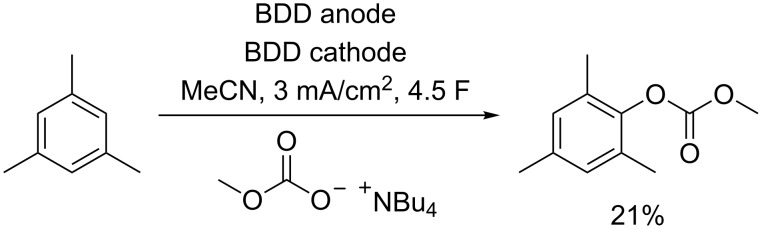
Direct generation of mesityl methyl carbonate by dehydrogenative functionalization.

Electrolysis experiments were conducted in undivided 5 mL beaker-type cells. Initial studies with benzene as the aromatic compound in acetonitrile in the presence of the described methyl carbonate salt did not result in the desired organic carbonate. Polymerization of the benzene was most likely and quinoide products were generated due to water traces in the commercially available acetonitrile. In order to investigate the potential functionalization of a side chain, xylene was tested. In contrast to benzene, traces of two compounds were detected by GC–MS analysis with a matching molar mass for a mono-functionalized product. A comparison with reference material revealed that these signals refer to the core and side-chain functionalization of xylene with a strong preference for the initially targeted functionalization at the core. However, only traces were observed, and no material could be isolated. We assumed that the presence of different aromatic positions lowered the selectivity of the reaction. We changed to mesitylene as the arene, since it exhibits equivalent aromatic positions for carbonate functionalization. This approach enabled a selective process and led to sufficient conversion for further studies. By optimization studies, we were able to produce 21% of the organic carbonate according to ^1^H NMR analysis on a 0.5 mmol scale (Scheme 2). Similar to the conversion of xylene, we also detected a weak signal of the corresponding side-chain functionalized product. Within our studies, we observed that only acetonitrile as solvent and BDD as anode material led to a successful conversion. Other electrodes and solvents (see Supporting Information File 1) indicated no traces of product in accordance with the high potential range accessible with this electrolyte–electrode combination. Loss of material occurs due to oligomerization of mesitylene and the co-generation of mesityl aldehyde and mesityl acetamide.

**Figure 2:**
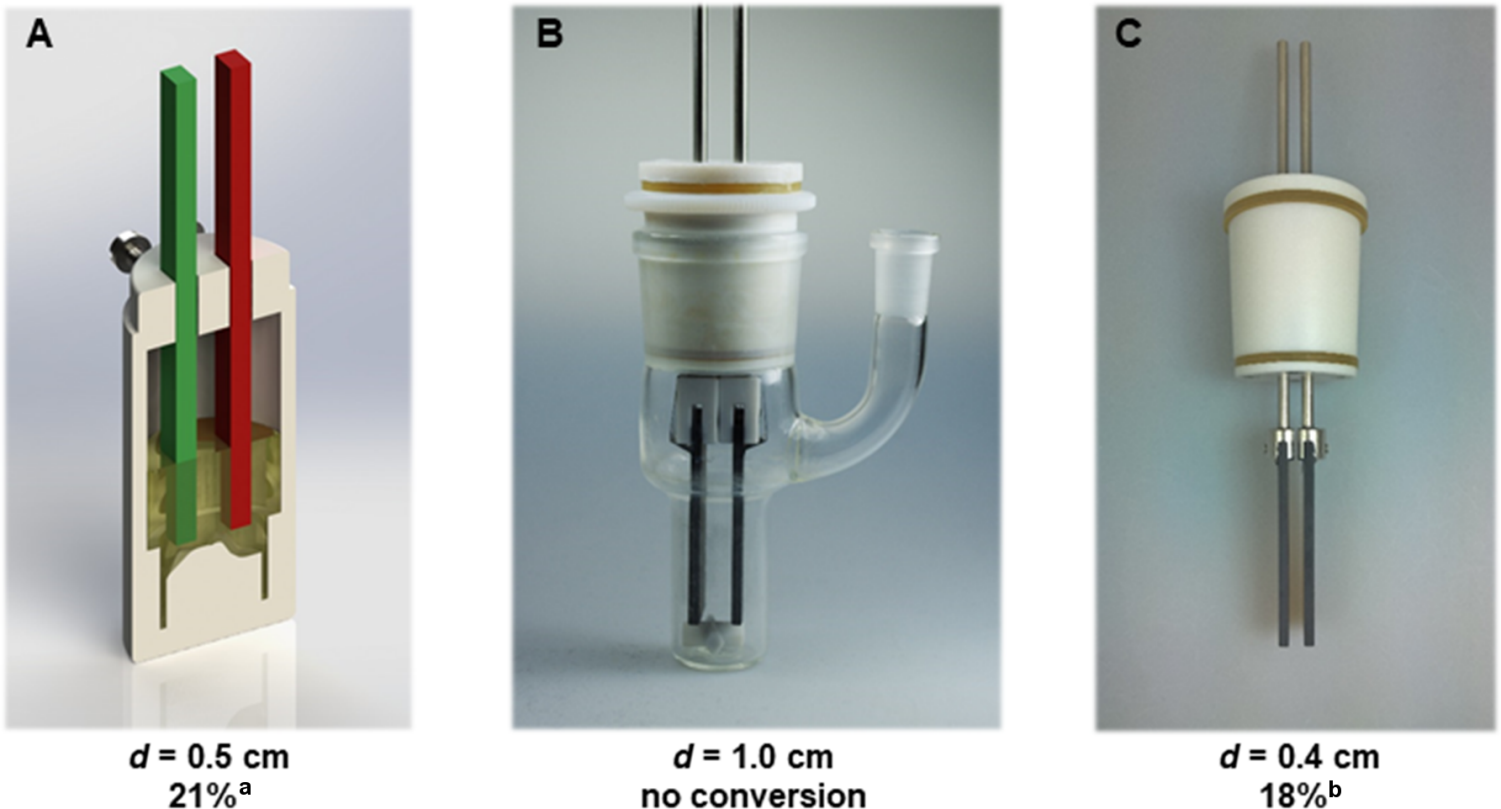
Influence of the electrode distance; ^a 1^H NMR yield; ^b^isolated yield.

According to cyclovoltammetric measurements (see Supporting Information File 1), it is most likely that mesitylene is oxidized prior to the tetrabutylammonium methyl carbonate. However, oxidation potentials are close to each other, which might lower the efficienty of the conversion. Tetrabutylammonium methyl carbonate serves as nucleophile and supporting electrolyte in this system. Although this unification is a straightforward approach, it can complicate aspects like optimization. Variation of the concentration revealed that only a very small concentration window (≈0.1 M) enables product generation in our set-up. Increased concentrations of the carbonate nucleophile, which might be beneficial at first sight, led to no conversion. Similar results emerged within scale-up, when a significant effect of the electrode distance occurred (Figure 2).

To generate a sufficient amount of material for work-up studies, we conducted conversions in 25 mL beaker-type cells. However, neither product formation nor conversion were observed. Since all parameters were constant except the electrode distance, which increased from 0.5 cm (set-up A) to 1.0 cm (set-up B), we repeated these experiments with a lower electrode distance of 0.4 cm (set-up C, Figure 2). This latter variation afforded selective conversion and an isolated yield of 18%. Separation of the product was achieved with a short-path distillation. A possible rationale for these effects is the sensitive influence of the carbonate nucleophile, which serves as nucleophile and supporting electrolyte. However, this behaviour is currently inexplicable for us and its elucidation is still in progress.

Within our studies on a potential scope of aromatic compounds, the symmetric 1,3,5-threefold substitution pattern showed best selectivity according to GC–MS measurements. Since the nucleophilic attack is controlled by sterics, non-symmetric substitution patterns gave weak signals and product mixtures. Generally, the nucleophilicity of carbonates is limited and therefore, the choice of suitable arenes is crucial. Heterofunctionalizations like fluoro, chloro, and methoxy groups are generally accepted, but it depends on the substitution pattern. The sensitive interplay of inefficient oxidation at highly positive oxidation potentials and the oligomerization tendency of electron-rich arenes limit the scope (see Supporting Information File 1).

## Conclusion

The first direct electrochemical generation of organic carbonates by dehydrogenative coupling at arenes was established. Even though this ambitious method is currently restricted to mesitylene, efforts are being made to develop an electrolysis protocol, which allows better conversions and higher yields. Nevertheless, the present results indicate that with BDD anodes this electro-conversion should be feasible in a general context, and might open the door to further direct installation of oxygen functionalization onto aromatic substrates.

## Supporting Information

File 1 Synthesis protocols, analytical data, GC chromatograms, MS spectra, and NMR spectra.
