# Supplementary material for: Direct electrochemical generation of organic carbonates by dehydrogenative coupling
Source: Beilstein J Org Chem. 2018 Jun 27;14:1578–82. doi: 10.3762/bjoc.14.135 (PMC6037012; doi:10.3762/bjoc.14.135)
Supplement: File 1 — Synthesis protocols, analytical data, GC chromatograms, MS spectra, and NMR spectra. [file Beilstein_J_Org_Chem-14-1578-s001.pdf]

Supporting Information

for

Direct electrochemical generation of organic carbonates by dehydrogenative coupling

Tile Gieshoff[1,2], Vinh Trieu[3], Jan Heijl[4] and Siegfried R. Waldvogel*[1,2]

Address: [1]Institute of Organic Chemistry, Johannes Gutenberg University Mainz, Duesbergweg 10-14, 55128 Mainz, Germany, [2]Graduate School Materials Science in Mainz, Staudingerweg 9, 55128 Mainz, Germany, [3]Covestro AG, Kaiser-Wilhelm-Allee 60, 51373 Leverkusen, Germany, [4]Covestro NV, Haven 507 - Scheldelaan 420, 2040 Antwerpen, Belgium

Email: Siegfried R. Waldvogel - waldvogel@uni-mainz.de

* Corresponding author

Synthesis protocols, analytical data, GC chromatograms, MS spectra, and NMR spectra

Table of Contents

General information S2

Synthesis protocols S2

Parameter screening and scope studies S3

Cyclovoltammetric studies S11

NMR spectra S12

References S14

General information

All reagents were used in analytical grades and were obtained from commercial sources. Solvents were purified by standard methods.[1]

Gas chromatography was performed on a Shimadzu GC-2025 (Shimadzu, Japan) using a Zebron ZB-5MSi column (Phenomenex, USA; length: 30 m, inner diameter: 0.25 mm, film: 0.25 µm, pre-column: 5 m, carrier gas: hydrogen). GC-MS measurements were carried out on a Shimadzu GC-2010 (Shimadzu, Japan) using a Zebron ZB-5MSi column (Phenomenex, USA; length: 30 m, inner diameter: 0.25 mm, film: 0.25 µm, pre-column: 5 m, carrier gas: helium) combined with a GCMS-QP2010.

Spectroscopy and spectrometry: ^{1}H NMR and ^{13}C NMR were recorded at 25 °C by using a Bruker Avance II 400 or a Bruker Avance III HD 400 (Analytische Messtechnik, Karlsruhe, Germany). Chemical shifts (*δ*) are reported in parts per million (ppm) relative to TMS as internal standard or traces of $CHCl_3$ (^{1}H 7.26 ppm, ^{13}C 77.16 ppm) in the corresponding deuterated solvent.[2] Mass spectra and high resolution mass spectra were obtained by using a Agilent 6545 Q-ToF MS apparatus employing ESI+.

Instrumentation and Electrode Materials: The undivided cells are made of Teflon and were used for the screening experiments.[3] For the electrochemical reactions the following electrode materials were used: glassy carbon (SIGRADUR® G, HTW, Thierhaupten, Germany), boron-doped diamond (DIACHEM®, 15 µm boron-doped diamond layer on 3 mm silicon support (geometrical surface area does not include additional 60% surface area arising from flatness imperfection of the surface), CONDIAS GmbH, Itzehoe, Germany), isostatic graphite (SIGRAFINE® V2100, SGL Carbon, Bonn /Bad Godesberg, Germany) and platinum (99.9% Pt, ÖGUSSA GmbH, Wien, Austria). As power source a multi-channel galvanostat was employed. This set-up can be commercially purchased from IKA-Werke GmbH & CO KG, Staufen, Germany, as IKA Screening System.

Cyclovoltammetric studies: Cyclic voltammetry was performed with a Metrohm 663 VA stand equipped with a *µ*-Autolab type III potentiostat (Metrohm AG, Herisau, Switzerland). WE: boron-doped diamond electrode tip, 2 mm in diameter; CE: glassy carbon rod; RE: Ag/$AgNO_3$ (silver wire in 0.1 M Bu_4NBF_4/CH_3CN, 0.01 M $AgNO_3$, E_0 measured as 97 mV vs. FcH/FcH+). Values are internally referenced vs. ferrocene. Scan rate = 100 mV/s.

Synthesis protocols

Tetrabutylammonium methyl carbonate

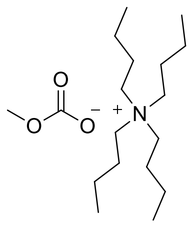

29.3 g (22.5 mmol) of a commercially available 21 wt% solution of tetrabutylammonium methoxide in methanol was placed in a round-botton flask and equipped with a gas injection tube. Carbon dioxide (4.5 purity) was passed through the solution vigorously for four hours. The solvent was removed under reduced pressure. For complete removal of residual methanol, the resulting viscous oil was evaporated by short path distillation in high vacuum. The resulting solid was dried overnight with high vacuum and yielded 5.2 g (16.4 mmol; 73%) colorless material. Analytical results were in accordance with the reported ones.[4]

^{1}H NMR (400 MHz, $CDCl_3$) δ = 3.36 (s, 3H), 3.24–3.17 (m, 8H), 1.58–1.46 (m, 8H), 1.30 (h, J = 7.3 Hz, 8H), 0.86 (t, J = 7.3 Hz, 12H).

^{13}C NMR (101 MHz, $CDCl_3$) δ = 158.27, 58.72, 52.12, 23.98, 19.67, 13.61.

Mesityl methyl carbonate

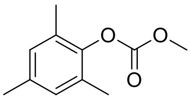

In an undivided 25 mL beaker-type glas cell, a solution of 2 mmol mesitylene and 2 mmol tetrabutylammonium methyl carbonate (635 mg) in 20 mL acetonitrile (278 µL) is electrolyzed with a current density of 3 mA/cm^2 and an electrode distance of 0.4 cm until 4.5 F charge is applied. Boron-doped diamond (surface area is 5 cm^2) the geometrical is used as anode and cathode material. After electrolysis, the electrolysis mixture is placed in a separation funnel and separated with 50 mL ethyl acetate and 50 mL water. The organic layer is washed with 50 mL water and the dried with $MgSO_4$. The solvent is removed under reduced pressure and the product is separated by short-path distillation. 71 mg product (0.37 mmol, 18%) are obtained as colorless liquid.

^{1}H NMR (400 MHz, $CDCl_3$) δ = 6.87 (s, 2H), 3.91 (s, 3H), 2.27 (s, 3H), 2.16 (s, 6H).

^{13}C NMR (101 MHz, $CDCl_3$) δ = 153.76, 146.15, 135.59, 129.66, 129.32, 55.49, 20.75, 15.93.

HRMS for $C_{11}H_{14}O_3$ (ESI+) $[M+H]^+$: calc.: 195.1021, found: 195.1026.

Parameter screening and scope studies

General procedure A for electrolysis in 5 mL Teflon cells for parameter screening and scope studies

Undivided 5 mL Teflon electrolysis cells were used. A solution of 0.5 mmol arene and 0.5 mmol tetrabutylammonium methyl carbonate in 5 mL acetonitrile is electrolyzed, until a defined amount of charge are applied. The detailed electrolysis parameters depend on the parameter screened.

For GC-MS analysis, approximately 0.5 mL of the electrolysis mixture is passed through a short silica plug with ethyl acetate as eluent and analyzed by GC-MS.

For quantification by NMR, the electrolysis mixture is placed in a separation funnel and separated with 20 mL water and 20 mL ethyl acetate. The organic layer is washed with

20 mL water and dried with $MgSO_4$. The solvent is removed under reduced pressure. A defined amount of 2,4,6-triiodophenol is added (5–15 mg). The whole mixture is dissolved in $CDCl_3$ and a proton NMR spectrum is recorded with an enhanced relaxation delay time of 20 s for quantification.

Tested anode materials

Boron-doped diamond, isostatic graphite, glassy carbon

Tested solvents

Acetonitrile, 1,1,1,3,3,3-hexafluoroisopropanol, methanol, sulfolan, *N*,*N*-dimethylformamide, methanol, 1,1-dimethylethanol, propylene carbonate

Substrate scope

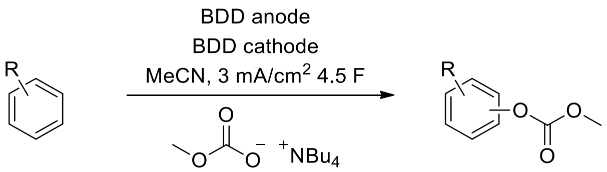

Product signals in GC-MS analysis with following arenes:

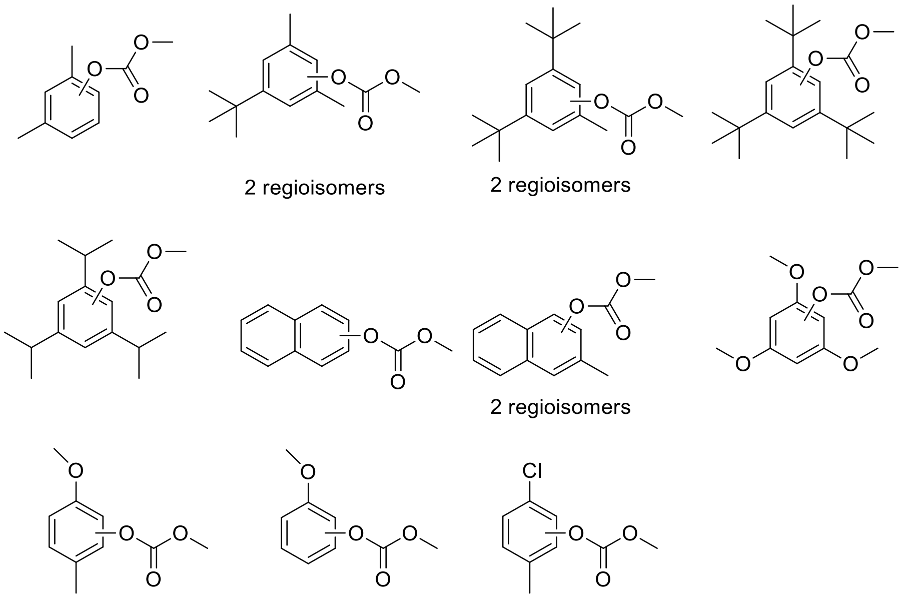

No product signals in GC-MS with following arenes:

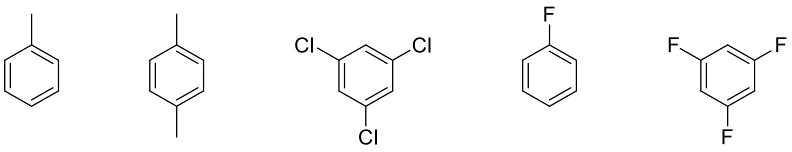

GC chromatograms and MS spectra for product traces:

x-axis displays the retention time (min) and y-axis the counts based on the MS instrument

Xylene

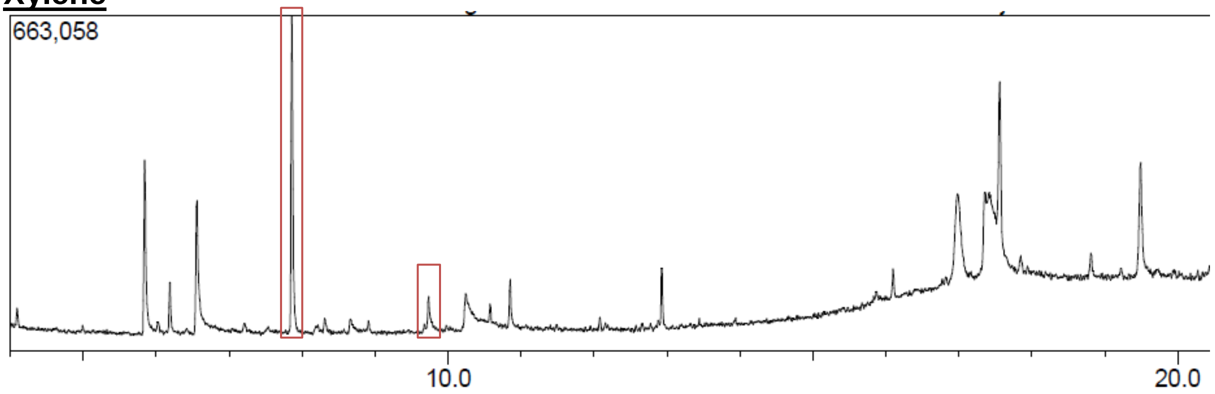

MS spectrum for the signal at 7.9 min

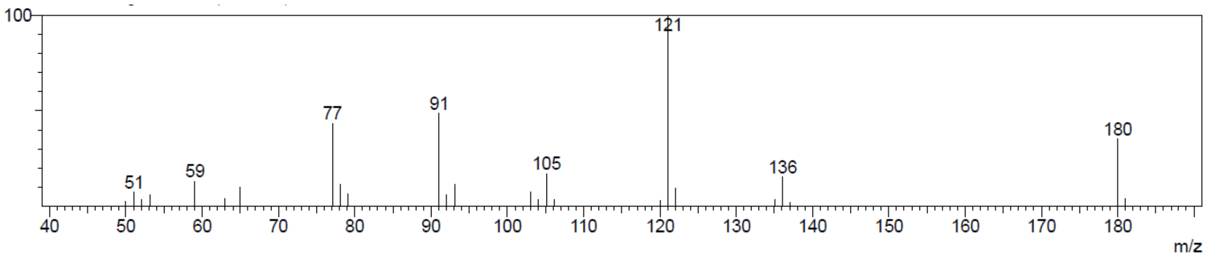

MS spectrum for the signal at 9.7 min

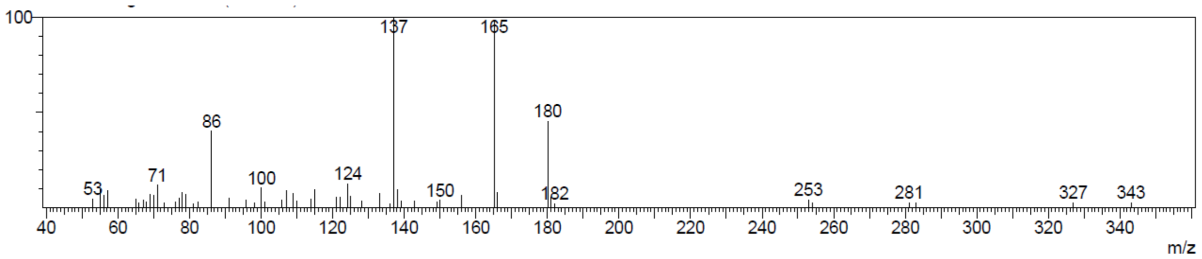

3,5-Dimethyl-1-(1,1-dimethylethyl)benzene

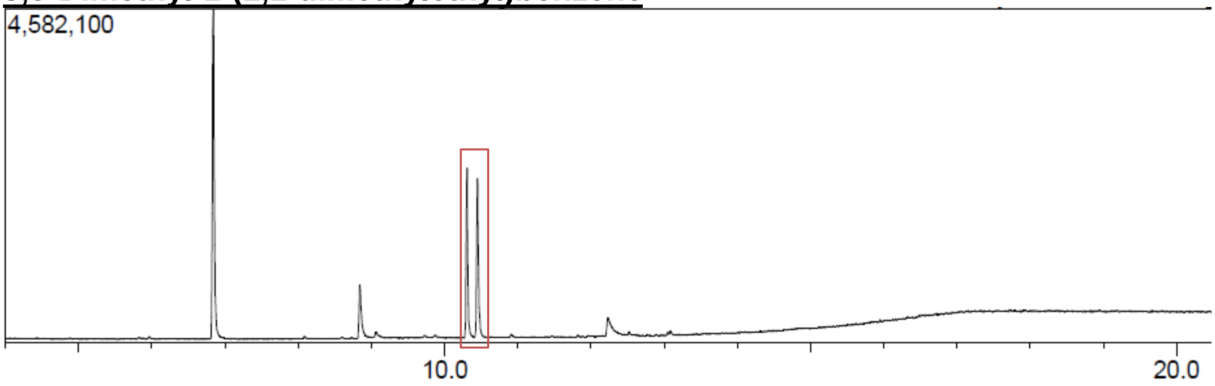

MS spectrum for the signal at 10.3 min

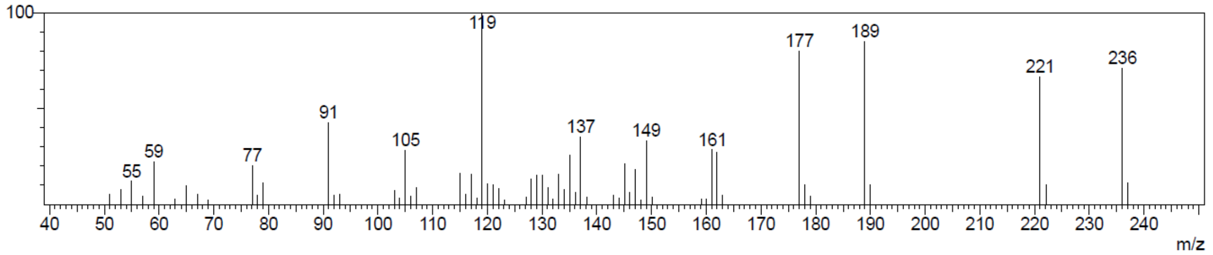

MS spectrum for the signal at 10.5 min

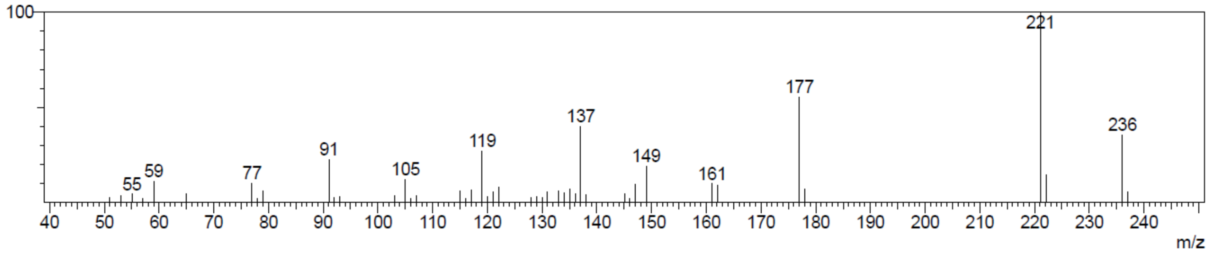

5-Methyl-1,3-bis(1,1-dimethylethyl)benzene

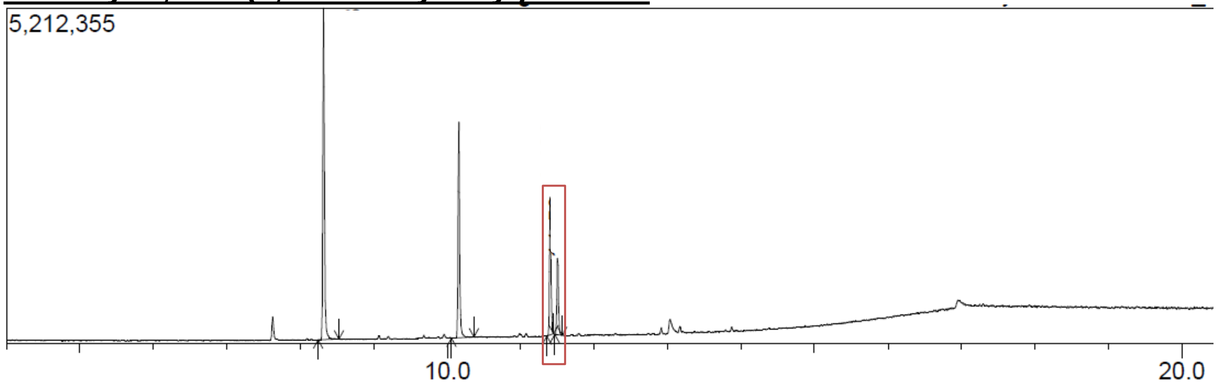

MS spectrum for the signal at 11.4 min

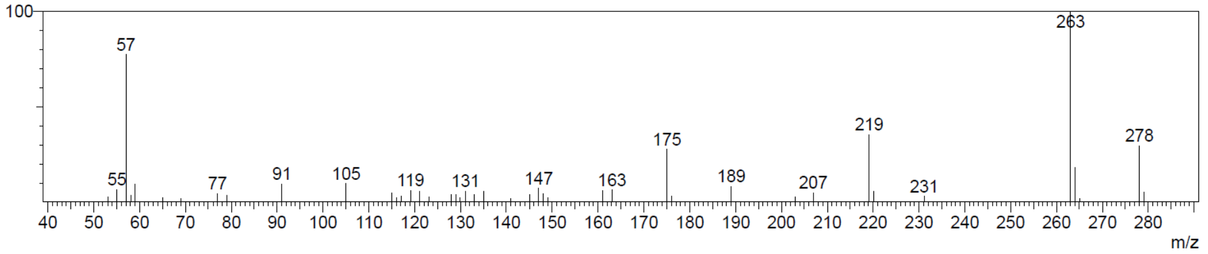

MS spectrum for the signal at 11.5 min

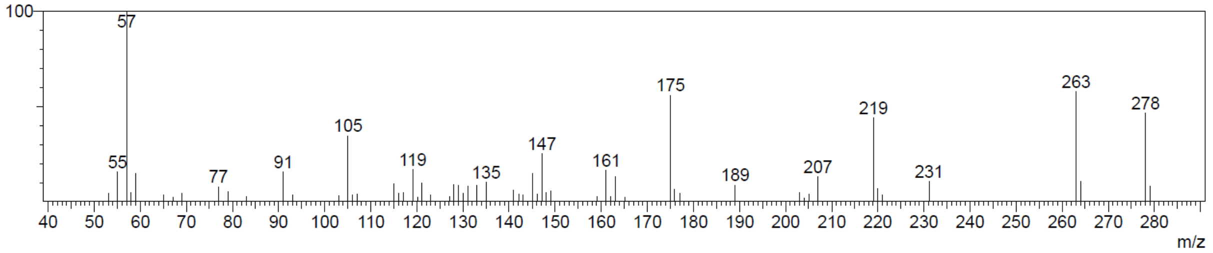

1,3,5-Tris(1,1-dimethylethyl)benzene

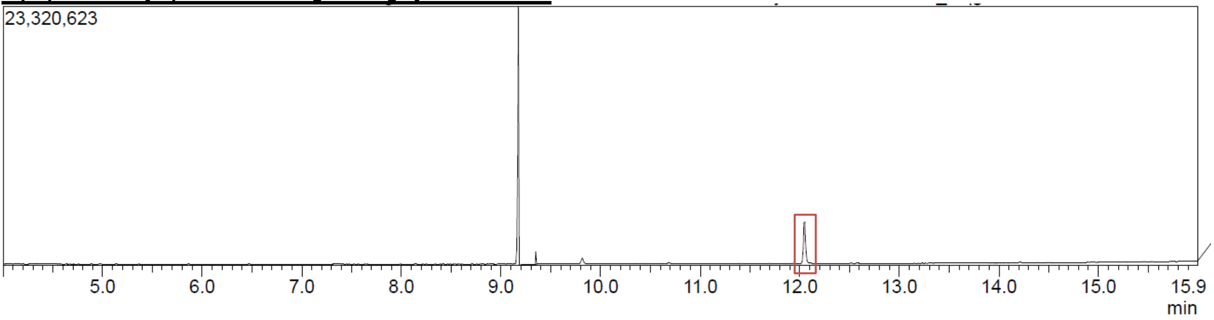

MS spectrum for the signal at 12.1 min

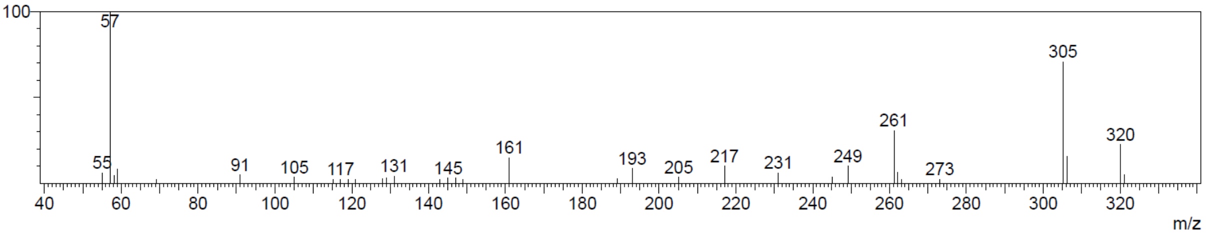

1,3,5-Tris(1-methylethyl)benzene

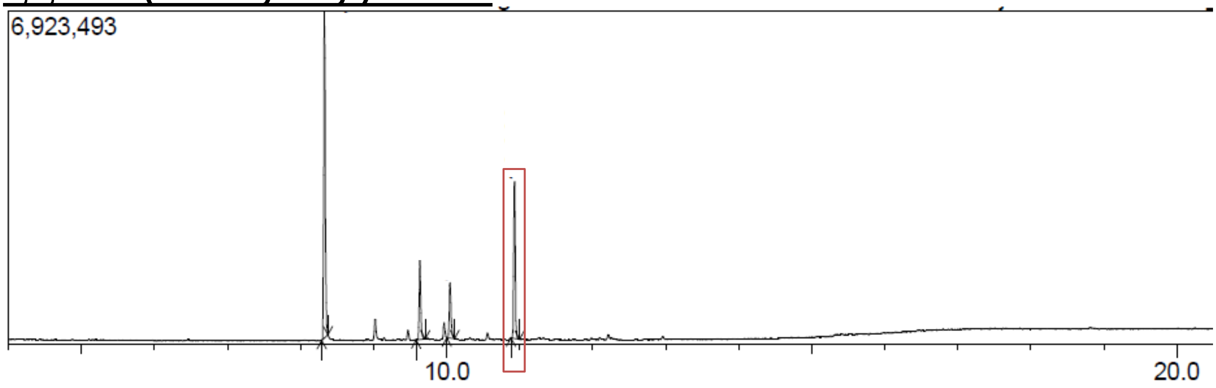

MS spectrum for the signal at 10.9 min

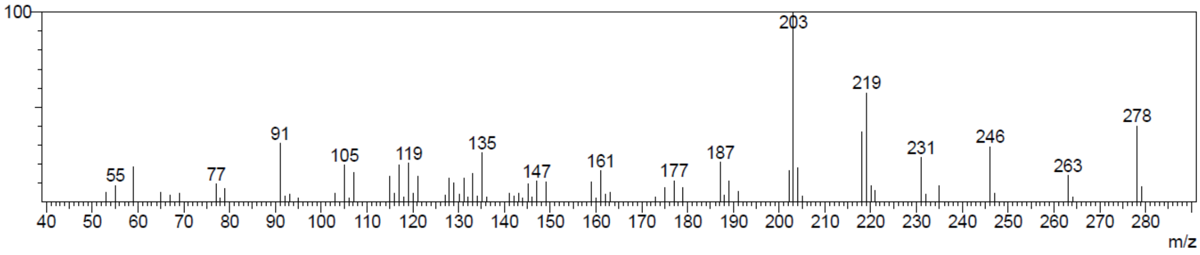

Naphthalene

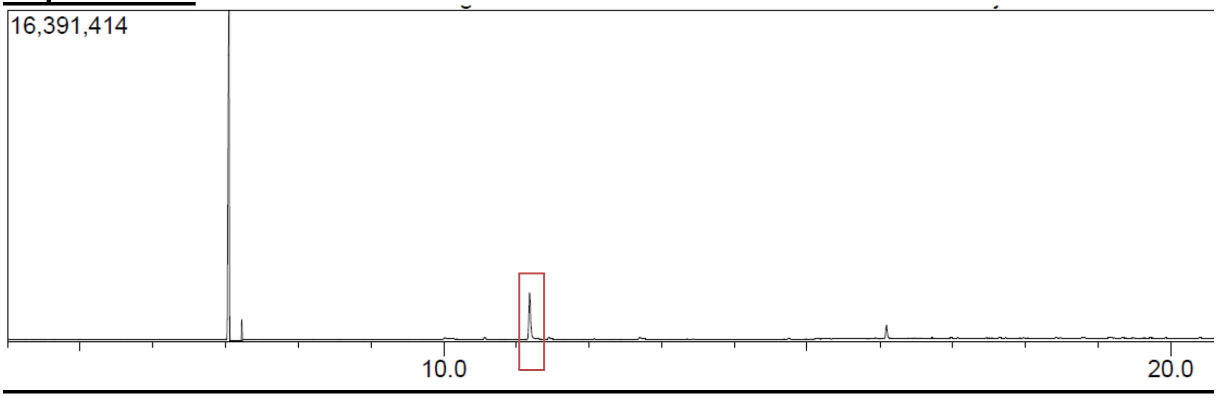

MS spectrum for the signal at 11.1 min

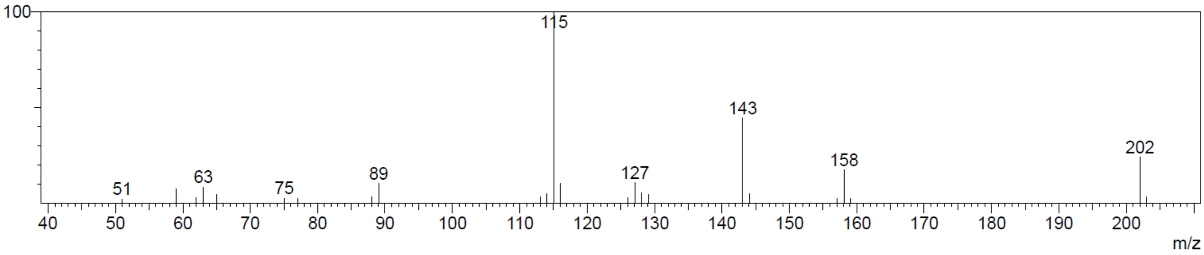

2-Methylnaphthalene

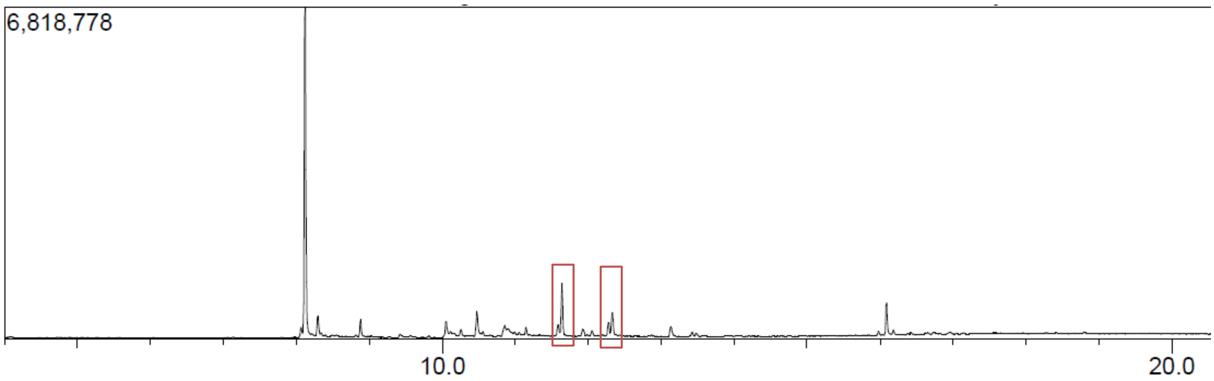

MS spectrum for the signal at 11.6 min

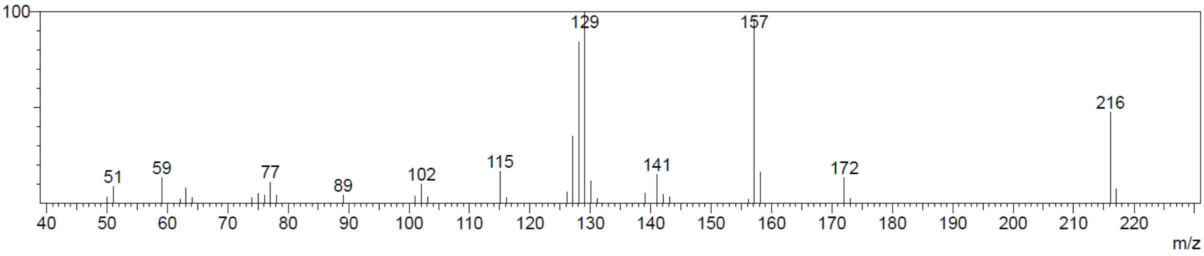

MS spectrum for the signal at 12.3 min

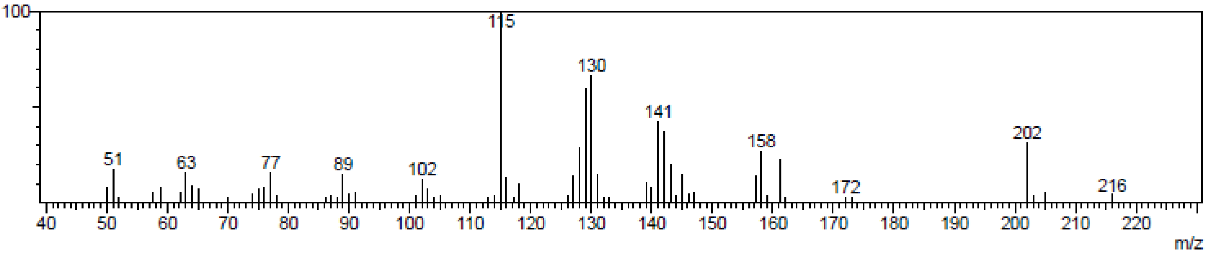

Trimethoxybenzene

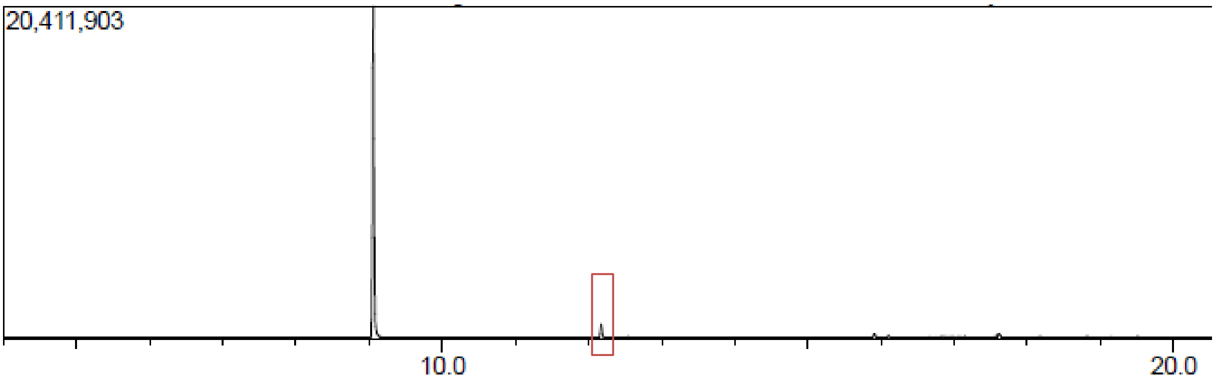

MS spectrum for the signal at 12.2 min

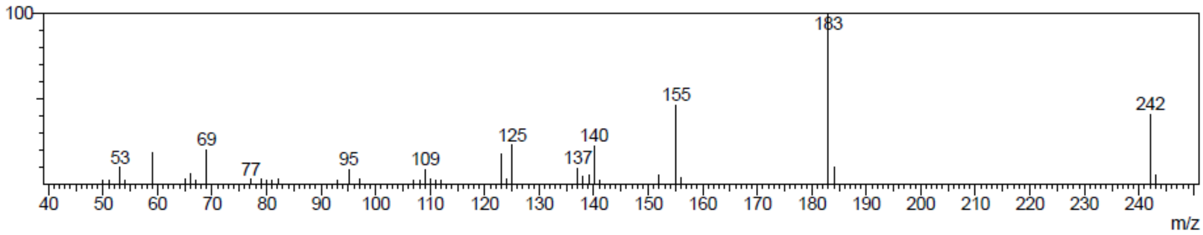

4-Methylanisole

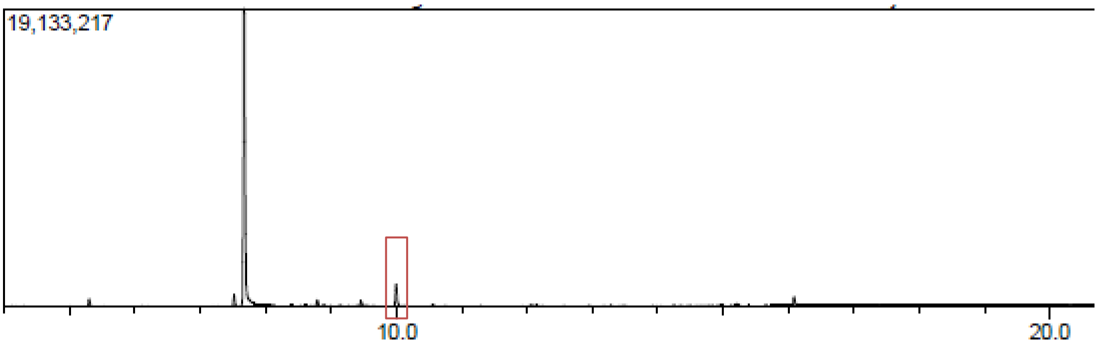

MS spectrum for the signal at 10.0 min

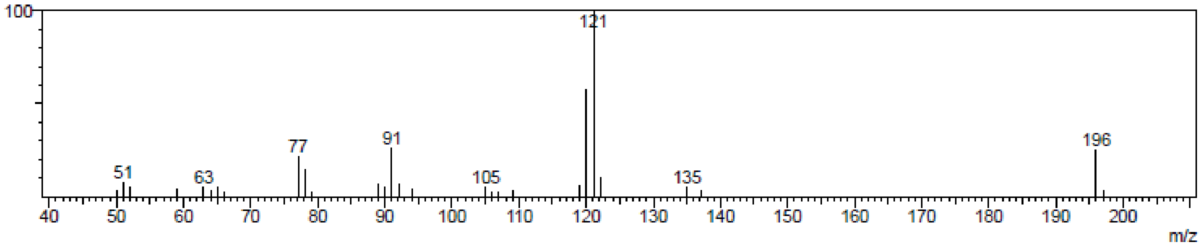

Anisole

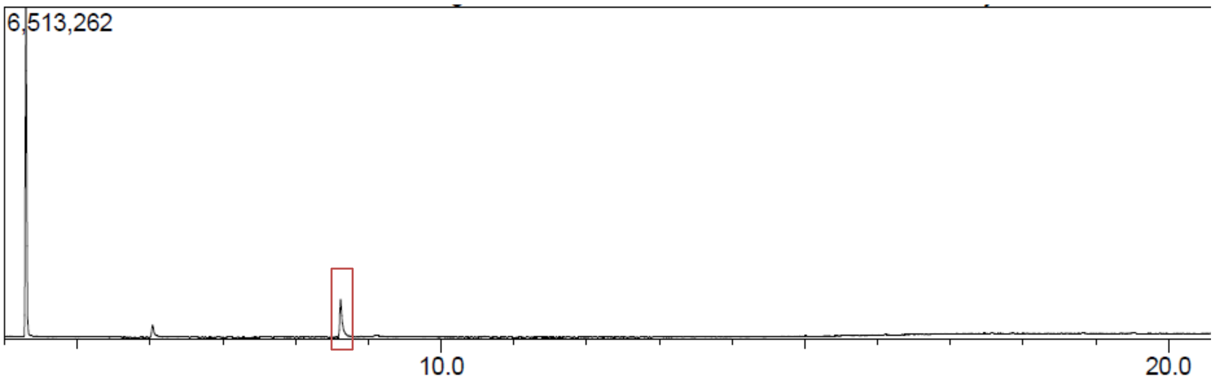

MS spectrum for the signal at 8.6 min

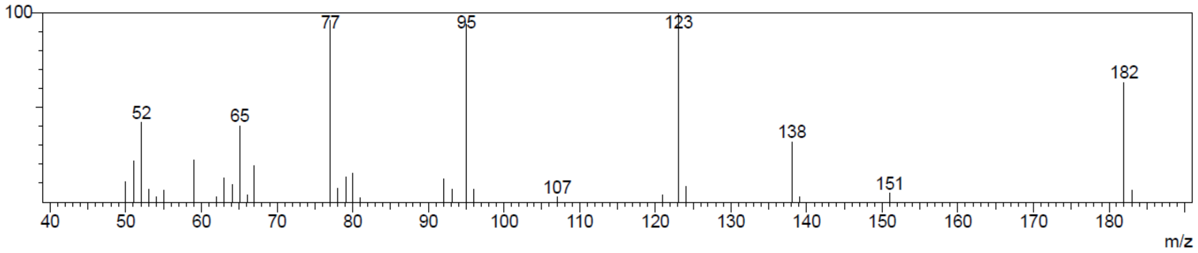

4-Methylchlorobenzene

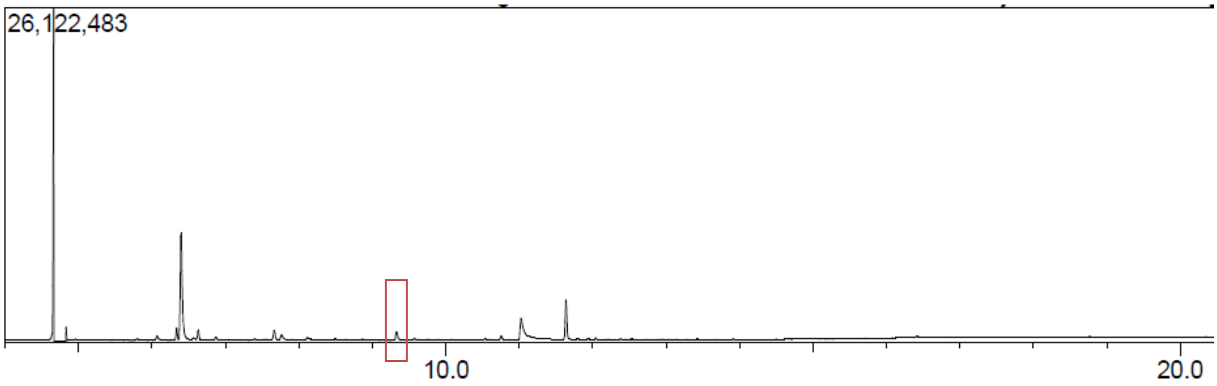

MS spectrum for the signal at 9.3 min

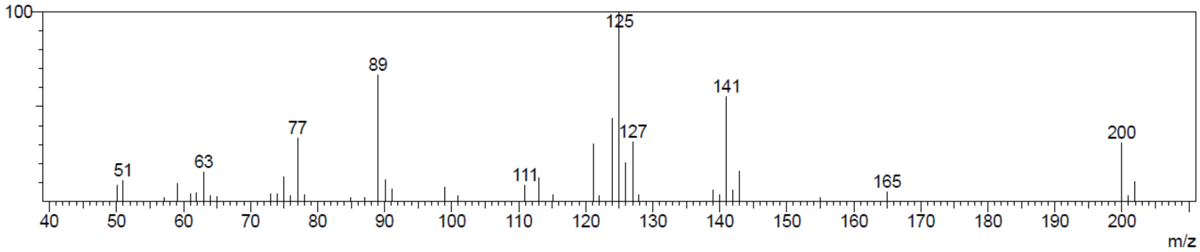

Cyclovoltammetric studies

The cyclic voltammogramm shows the oxidation potential of mesitylene at 1.75 V in a NBu_4BF_4 solution in acetonitrile (Figure 1; black curve). In the tetrabutylammonium methyl carbonate containing electrolye, a electrolyte decomposition starting at approximately 1.4 V can be observed (Figure 2; red curve)). Upon addition of mesitylene, decomposition is suppressed and a slight increase of current can be observed as an overlap of mesitylene and electroyte oxidation (Figure 2; black curve). This behaviour implies that mesitylene is oxidized first.

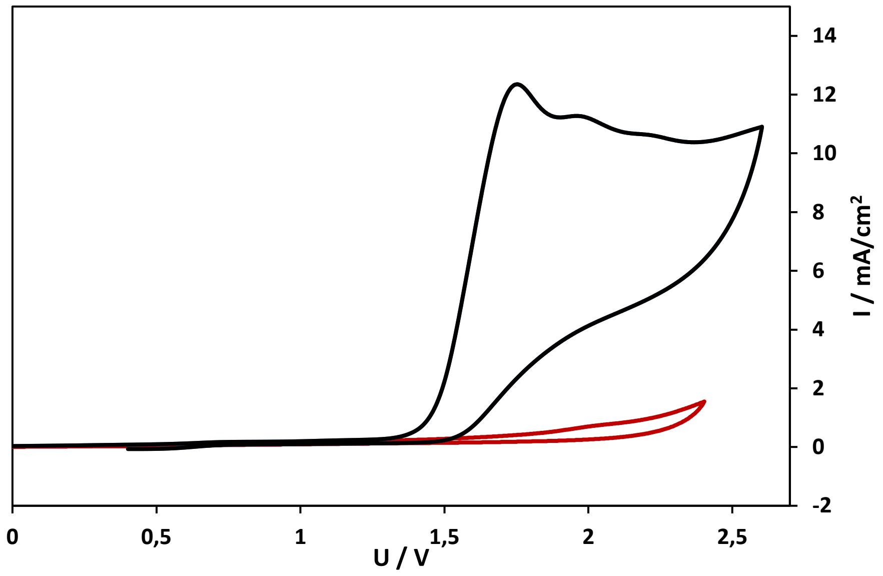

Figure 1: Cyclovoltammogram of mesitylene (0.05 mmol; 7 µL) in a 0.1 M solution of NBu_4BF_4 in acetonitrile. The test measurement without mesitylene is shown in red.

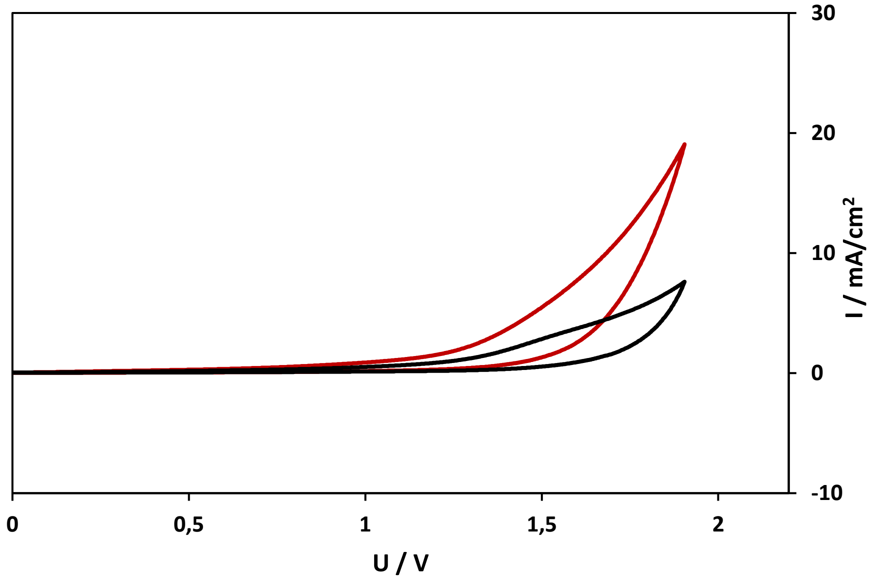

Figure 2: Cyclovoltammogram of mesitylene (0.05 mmol; 7 µL) in a 0.1 M solution of tetrabutylammonium methyl carbonate in acetonitrile. The test measurement without mesitylene is shown in red.

NMR spectra

Tetrabutylammonium methyl carbonate

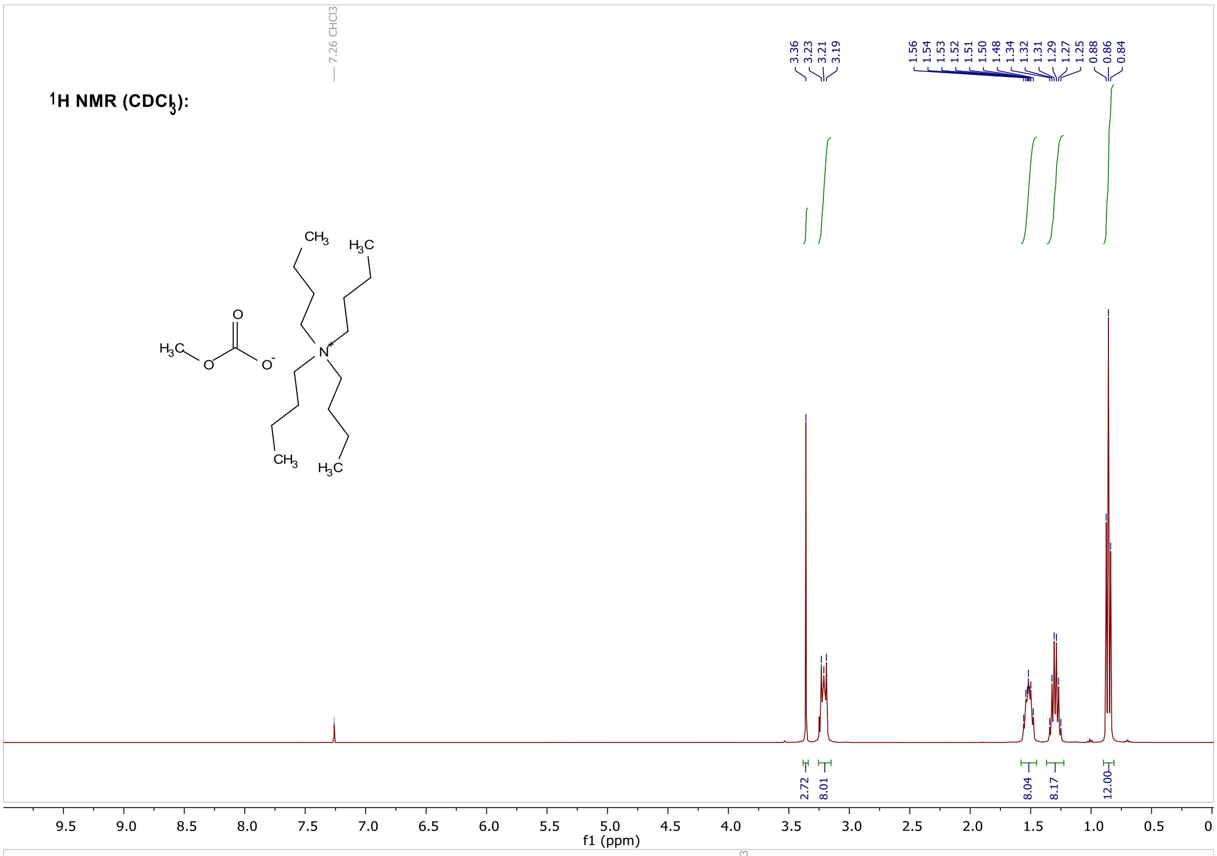

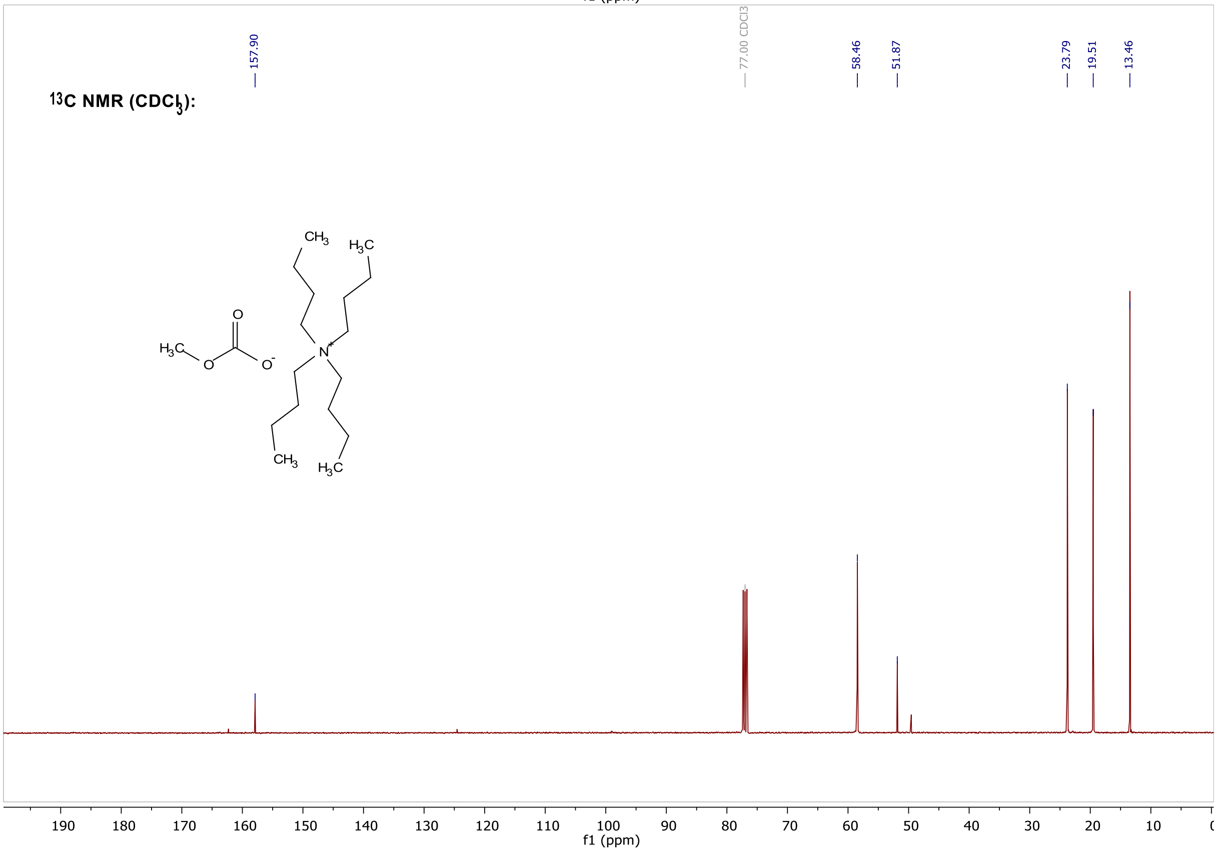

Mesityl methyl carbonate

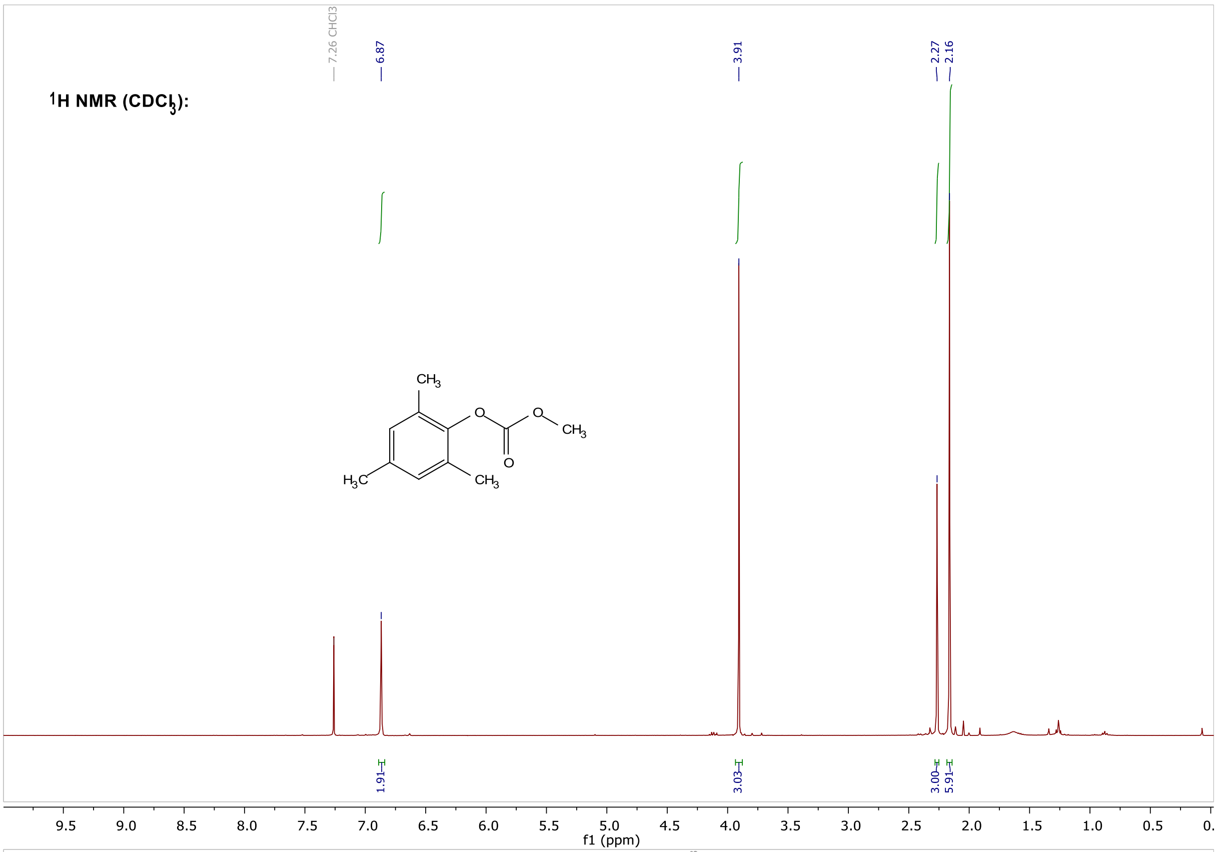

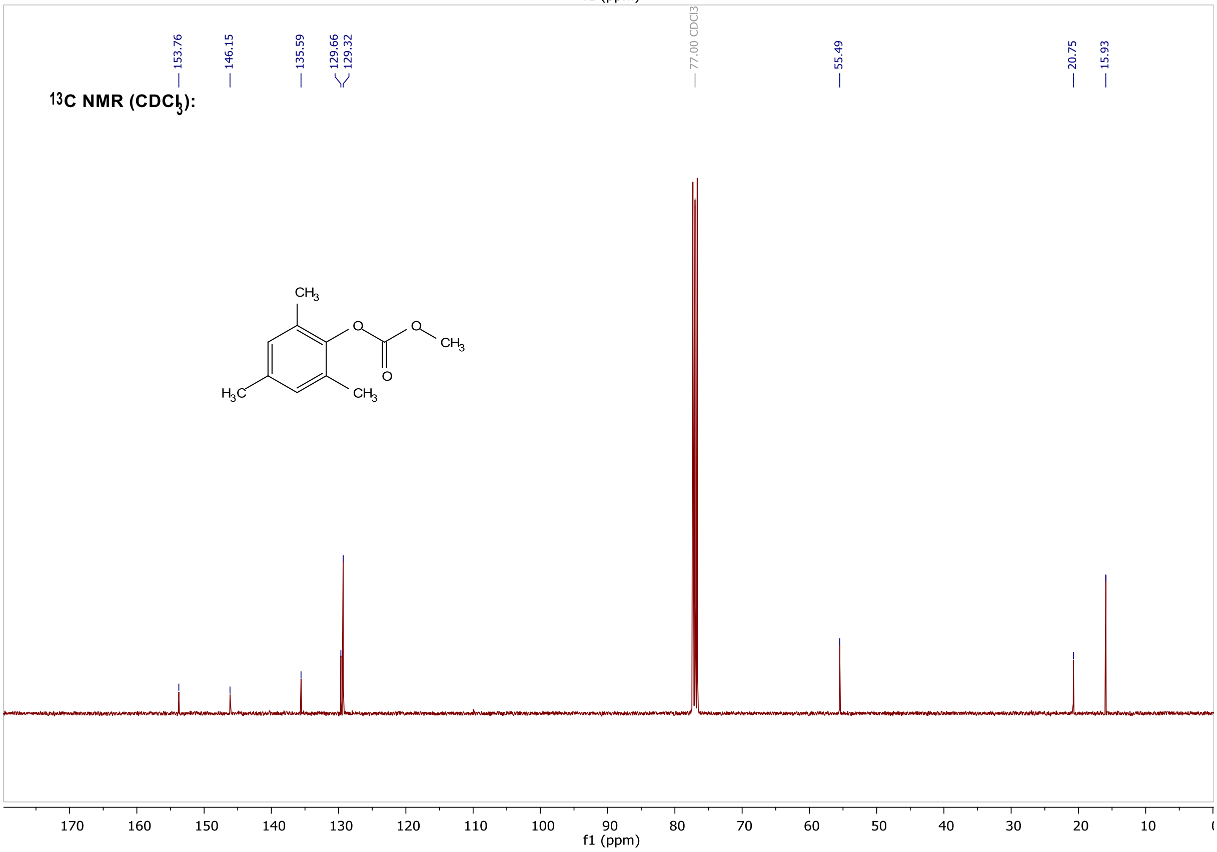

References

[1] Armarego, W. L. F.; Chai, C. L. L. *Purification of laboratory chemicals*, Butterworth-Heinemann, Amsterdam, 5th ed., **2003**.

[2] Berger, S.; Braun, S.; Kalinowski, H.-O. *NMR-Spektroskopie von Nichtmetallen*, Thieme, Stuttgart, **1994**.

[3] Gütz, C.; Klöckner, B.; Waldvogel, S. R. *Org. Process Res. Dev.* **2016**, *20*, 26–32.

[4] Verdecchia, M.; Feroci, M.; Palombi, L.; Rossi, L. *J. Org. Chem.* **2002**, *67*, 8287–8289
